# Successful HCV Therapy Reduces Liver Disease Severity and Inflammation Biomarkers in HIV/HCV-Coinfected Patients With Advanced Cirrhosis: A Cohort Study

**DOI:** 10.3389/fmed.2021.615342

**Published:** 2021-02-01

**Authors:** Luz Maria Medrano, Juan Berenguer, Sergio Salgüero, Juan González-García, Cristina Díez, Víctor Hontañón, Pilar Garcia-Broncano, Luis Ibañez-Samaniego, José M. Bellón, María Angeles Jiménez-Sousa, Salvador Resino

**Affiliations:** ^1^Unidad de Infección Viral e Inmunidad, Centro Nacional de Microbiología, Instituto de Salud Carlos III, Madrid, Spain; ^2^Unidad de Enfermedades Infecciosas/VIH, Hospital General Universitario “Gregorio Marañón”, Madrid, Spain; ^3^Instituto de Investigación Sanitaria del Gregorio Marañón, Madrid, Spain; ^4^Unidad de Análisis Clínicos, Hospital Universitario Fundación Alcorcón, Alcorcón, Spain; ^5^Unidad de VIH, Servicio de Medicina Interna, Hospital Universitario “La Paz”/IdiPAZ, Madrid, Spain; ^6^Ragon Institute of MGH, MIT and Harvard, Cambridge, MA, United States; ^7^Servicio de Aparato Digestivo, Hospital General Universitario “Gregorio Marañón”, Madrid, Spain; ^8^Fundación para la Investigación Biomédica, Hospital General Universitario Gregorio Marañón, Instituto de Investigación Sanitaria Gregorio Marañón (IiSGM), Madrid, Spain

**Keywords:** chronic hepatitis C (CHC), direct-acting antiviral (DAA) therapy, HIV/HCV co-infected patients, cirrhosis, inflammation, coagulopathy, angiogenesis

## Abstract

**Background:** Eradication of hepatitis C virus (HCV) promotes an improvement in liver disease and the deactivation of the immune system. Here, we aimed to evaluate the changes in liver disease scores and plasma biomarkers following HCV clearance with direct-acting antivirals (DAAs) in HIV-infected patients with advanced HCV-related cirrhosis.

**Methods:** We performed an observational study of 50 patients with advanced cirrhosis who received DAAs therapy. Variables were assessed at baseline and 48 weeks after HCV treatment completion. Epidemiological and clinical data were collected through an online form. Liver stiffness measurement (LSM), hepatic venous pressure gradient (HVPG), and Child-Pugh-Turcotte (CTP) were evaluated by physicians. Plasma biomarkers were measured by multiplex immunoassay.

**Results:** We found significant decreases in severity scores of liver disease [LSM (*q*-value < 0.001), HVPG (*q*-value = 0.011), and CTP (*q*-value = 0.045)] and plasma biomarkers [LBP (*q*-value < 0.001), IP-10 (*q*-value < 0.001), IL-8 (*q*-value < 0.001), IL-18 (*q*-value < 0.001), IL-1RA (*q*-value = 0.013), OPG (*q*-value < 0.001), sVCAM-1 (*q*-value < 0.001), sICAM-1 (*q*-value < 0.001), PAI-1 (*q*-value = 0.001), and VEGF-A (*q*-value = 0.006)]. We also found a significant direct association between the change in LSM values and the change in values of LBP (*q*-value < 0.001), IP-10 (*q*-value < 0.001), MCP-1 (*q*-value = 0.008), IL-8 (*q*-value < 0.001), IL-18 (*q*-value < 0.001), OPG (*q*-value = 0.004), sVCAM-1 (*q*-value < 0.001), sICAM-1 (*q*-value < 0.001), and PAI-1 (*q*-value = 0.002). For CTP values, we found significant positive associations with IP-10 (*q*-value = 0.010), IL-6 (*q*-value = 0.010), IL-1RA (*q*-value = 0.033), and sICAM-1 (*q*-value = 0.010).

**Conclusion:** The HCV eradication with all-oral DAAs in HIV/HCV-coinfected patients with advanced cirrhosis promoted an improvement in the severity of advanced cirrhosis and plasma biomarkers (inflammation, coagulopathy, and angiogenesis). The decrease in plasma biomarkers was mainly related to the reduction in LSM values.

## Introduction

Hepatitis C virus (HCV) infection triggers an immune response against the viral infection, but it also promotes chronic inflammation, immune activation, and immune dysfunction that accelerate the development of liver fibrosis and other comorbidities (1, 2). Additionally, in advanced stages, cirrhosis-associated immune dysfunction (CAID) usually appears, characterized by higher inflammation levels, immune activation, and immunosuppression in the liver and peripheral blood (3). The extent of CAID is directly related to liver disease severity and plays a decisive role in its progression to hepatic decompensation (3). Patients with hepatic decompensation may develop complications related to portal hypertension, such as ascites, jaundice, variceal bleeding, or hepatic encephalopathy, decreasing quality of life and survival rates (4). The direct-acting antiviral agents (DAAs) against HCV have increased sustained virologic response (SVR) rates in patients with advanced HCV-related cirrhosis, which improves the quality of life and reduce morbidity from cirrhosis (5). Additionally, significant decreases in liver disease scores [liver stiffness measurement (LSM), hepatic venous pressure gradient (HVPG) or Child-Pugh-Turcotte (CTP)] (6–14) and plasma biomarkers related to inflammation and immune activation (15–20) have been described in HCV-monoinfected patients after SVR with all-oral DAAs.

The human immunodeficiency virus (HIV) infection accelerates the course of chronic hepatitis C (CHC), resulting in higher rates of cirrhosis, end-stage liver disease, and death than HCV-monoinfected patients (21). HIV infection increases a series of negative factors such as HCV replication, hepatic inflammation, hepatocyte apoptosis, and microbial translocation, while it also leads to a deterioration of the specific immune responses against HCV (22, 23). Antiretroviral therapy (ART) delays the fibrosis progression and reduces clinical complications, but despite suppressive ART, HIV/HCV-coinfected patients still have abnormally high levels of plasma biomarkers related to bacterial translocation, immune activation, inflammation, and coagulation (23), which are related to higher morbidity and mortality (24). Similarly, DAA treatments against HCV infection also achieve elevated SVR rates (25, 26) and delay CHC progression in HIV/HCV-coinfected patients (27, 28). Several reports have shown a significant decrease in plasma biomarkers related to inflammation and immune activation after SVR with all-oral DAAs in HIV/HCV-coinfected patients (18, 29–33). However, DAA therapy alone does not entirely block uncontrolled inflammation and liver injury, particularly with advanced liver disease (34). Thus, some cirrhotic patients who achieve SVR with DAAs remain at risk of cirrhosis progression and developing hepatocellular carcinoma (1).

In this study, we aimed to evaluate the changes in liver disease scores and plasma biomarkers following HCV clearance with DAAs in HIV-infected patients with advanced HCV-related cirrhosis.

## Patients and Methods

### Patients

We carried out a multicenter observational study (repeated measures design) in 50 HIV-infected patients with advanced HCV-related cirrhosis who started anti-HCV therapy with all-oral DAAs. Patients were recruited at four tertiary referral hospitals in Madrid (Spain) between January 2015 and June 2016 (ESCORIAL study; see Appendix). The study was approved by the Ethics Committee of the Instituto de Salud Carlos III (CEI PI 41_2014) and was conducted according to the Declaration of Helsinki. All participants gave their written consent before enrollment.

The inclusion criteria were: (1) active HCV infection at baseline defined by detectable plasma HCV-RNA; (2) advanced cirrhosis [LSM ≥ 25 kPa, or HVPG ≥ 10 mmHg, or CTP ≥ 7, or prior history of liver decompensation (ascites, varices, bleeding esophageal, hepatic encephalopathy)]; (3) starting anti-HCV therapy with all-oral DAAs (without interferon (IFN) or ribavirin) and achieving an SVR defined as an undetectable HCV-RNA load 12 weeks after the finalization of anti-HCV treatment.

### Clinical Data and Samples

Clinical data and samples were collected at the baseline (initiation of HCV treatment) and at the end of follow-up (48 weeks after completing HCV therapy).

Epidemiological and clinical data were collected through an online form, which fulfilled the confidentiality requirements. These data were monitored to verify that all the information in the database matched the patients' records. The LSM was carried out by trained operators of transient elastography (FibroScan®, Echosens, Paris, France), as we previously described (35). LSM values were reported in kilopascals (kPa) and range from 2.5 to 75 kPa. The CTP score was calculated from five factors (total bilirubin, albumin, international normalized ratio, ascites, and encephalopathy) and range from 5 to 15 points. Hemodynamic studies were performed after overnight fasting under light sedation with intravenous midazolam, as we previously described (36). The HVPG was measured as the difference between wedged hepatic venous pressure and free hepatic venous pressure in millimeters of mercury (mmHg).

Blood samples were collected by venipuncture in EDTA tubes, which were then sent to the HIV HGM BioBank (http://hivhgmbiobank.com/?lang=en), where they were processed and immediately stored until use at −80°C.

### Enzyme-Linked Immunosorbent Assays

Plasma biomarkers were measured by ProcartaPlexTM multiplex immunoassay (Bender MedSystems GmbH, Vienna, Austria) by using a Luminex 200™ analyzer (Luminex Corporation, Austin, TX, United States) according to the manufacturer's specifications. Plasma biomarkers measured were: (i) *inflammation*: IFN-γ-inducible protein 10 (IP-10), monocyte chemoattractant protein-1 (MCP1), interleukin (IL)-8; IL-1β, IL-18, IL-6, tumor necrosis factor-alpha (TNF-α), interleukin-1 receptor antagonist (IL-1RA), osteoprotegerin (OPG) and soluble receptor activator of nuclear factor-kappa B ligand (sRANKL); (ii) *endothelial dysfunction*: soluble intercellular cell adhesion molecule 1 (sICAM-1); soluble vascular cell adhesion molecule 1 (sVCAM-1), and soluble tumor necrosis factor receptor 1 (sTNFR-1); (iii) *coagulopathy*: plasminogen activator inhibitor-1 (PAI-1) and D-dimer; (iv) *angiogenesis*: soluble receptor 1 for vascular endothelial growth factor (sVEGF-R1) and vascular endothelial growth factor A (VEGF-A). Because a high proportion of the analyzed samples were below the lower limit of detection, we used the raw fluorescence intensity values in arbitrary units, without subtracting blank, as a relative quantification of the analyte abundances (37).

We also used commercial simple ELISA assays for biomarkers that were not available by multiplex ELISA: Lipopolysaccharide binding protein (LBP) (R&D Systems, Minneapolis, MN, United States), soluble CD14 (sCD14) and fatty acid-binding protein 2 (FABP-2) (Raybiotech, Peachtree Corners, GA, United States). The lipopolysaccharide (LPS; Hycult Biotech, Uden, The Netherlands) was evaluated by a Limulus amebocyte lysate chromogenic endpoint ELISA.

### Statistical Analysis

Stata 15.0 (StataCorp, College Station, TX, United States) was used to perform statistical analyses. All *p*-values were two-tailed and were corrected for multiple testing by using the false discovery rate with Benjamini and Hochberg procedure (*q*-values), separately for the baseline and end of follow-up. The statistical significance was defined as *p* ≤ 0.05 or *q* ≤ 0.05, as appropriate.

Generalized Linear Mixed Models (GLMM) with a gamma distribution (log-link) was used to evaluate repeated measurements. Gamma distribution in GLMM is recommended for modeling skewed continuous outcomes. The log-link was applied only to the dependent variables (y), which were the severity scores of liver cirrhosis and plasma biomarkers. These outcome variables were analyzed separately, each one of them, so there was no collinearity problem. GLMM gives us the estimation of average increase [Δx = final value (x2) × baseline value (x1)] of each biomarker in each of the two times. GLMM models were also used to analyze the association between the change in plasma biomarkers (Δx) and the changes in severity scores of liver disease (Δy) during the follow-up. Due to the repeated measures design and the statistical analysis by GLMM, it was not necessary to adjust the models for covariates because each patient is their control. According to your positive or negative sign, this analysis gives us the regression coefficient (β), which indicates the effect's size and direction.

## Results

### Baseline Characteristics

Table 1 shows the characteristics of the 50 HIV/HCV-coinfected patients with advanced HCV-related cirrhosis. All patients were on ART before starting the study, and their plasma HIV viral load was undetectable (<50 copies/mL). None of the participants were actively using injection drugs.

**Table 1:** Summary of characteristics of HIV-infected patients with advanced HCV-related cirrhosis at baseline.

**Characteristics**	**Data**
No.	50
Gender (male)	39 (78%)
Age (years)	52.2 (48.8; 54.1)
Smoker	
Never	6 (12.2%)
Previously (≥6 months)	12 (24.5%)
Currently	31 (63.3%)
Alcohol intake	
Never	21 (42%)
Previously (≥6 months)	24 (48%)
Currently	5 (10%)
IVDU	
Never	12 (24%)
Previously (≥6 months)	38 (76%)
Currently	0 (0%)
Treatments	
Statins	8 (16%)
Previous IFNα therapy	23 (46%)
DAAs regimens	
Sofosbuvir + Ledipasvir	20 (40%)
Sofosbuvir + Daclatasvir	14 (28%)
Sofosbuvir + Daclatasvir + Simeprevir	3 (6%)
Sofosbuvir + Simeprevir	10 (20%)
Ombitasvir + Paritaprevir + Ritonavir + Dasabuvir	3 (6%)
Antiretroviral therapy	
NRTI + NNRTI-based	7 (14.3%)
NRTI + II-based	24 (49%)
NRTI + PI-based	6 (12.2%)
PI + II + others-based	4 (8.2%)
Others	8 (16.3%)
HIV markers	
Prior AIDS	18 (36%)
Nadir CD4^+^ T-cells (cells/mm^3^)	114.7 (70; 182)
Nadir CD4^+^ <200 cells/mm^3^	35 (76.1%)
CD4^+^ T-cells (cells/mm^3^)	439 (234; 721)
CD4+ <500 cells/mm^3^	30 (60%)
HCV markers	
HCV genotype	
1	32 (65.3%)
3	7 (14.3%)
4	10 (20.4%)
Log_10_ HCV-RNA (IU/mL)	6.2 (5.7; 6.7)
HCV-RNA ≥ 850.000 IU/mL	33 (66%)
Liver disease markers	
LSM (kPa)	31.6 (23.4; 40.7)
<25 kPa	16 (26.7%)
25–40 kPa	29 (48.3%)
≥40 kPa	15 (25%)
CTP	5 (5; 6)
CTP ≥ 7	6 (10.3%)
HVPG (mmHg)	16 (11; 18)
<16 mmHg	15 (48.4%)
16–20 mmHg	11 (35.5%)
≥20 mmHg	5 (16.1%)
Plasma biomarkers	
Bacterial translocation	
LPS (EU/mL)	0.9 (0.7; 1.2)
LBP (μg/mL)	1 (0.6; 1.4)
sCD14 (μg/mL)	2.1 (1.6; 3.2)
FABP-2 (ng/mL)	0.4 (0.1; 0.7)
Inflammation	
IP-10 (a.u.)	1,221.5 (865.3; 1,799.5)
MCP-1 (a.u.)	493.8 (276.1; 684.1)
IL-8 (a.u.)	117.8 (74.6; 185.8)
IL-1β (a.u.)	15 (13; 27.6)
IL-18 (a.u.)	988.5 (573.1; 1,670.9)
IL-6 (a.u.)	65.3 (33.8; 156.3)
TNF-α (a.u.)	9.8 (7.4; 12)
IL-1RA (a.u.)	18 (13; 25.9)
sRANKL (a.u.)	24.3 (19; 36.4)
OPG (a.u.)	217.5 (158.5; 307)
Endothelial dysfunction	
sVCAM-1 (a.u.)	10,747 (8,616.8; 12,142.3)
sICAM-1 (a.u.)	107.8 (63.5; 163.1)
TNFR-I (a.u.)	32 (20.4; 57.8)
Coagulopathy	
PAI-1 (a.u.)	1,054.8 (808.6; 1,315.3)
D-dimer (a.u.)	1,767.5 (653.4; 4,289.9)
Angiogenesis	
VEGF-A (a.u.)	76 (57.6; 90.8)
sVEGF-R1 (a.u.)	44.5 (32.4; 69.5)

*Statistics: Values are expressed as absolute number (percentage) and median (interquartile range)*.

*HCV, hepatitis C virus; HIV, human immunodeficiency virus; IVDU, intravenous drug user; IFNα, interferon-alpha; DAAs, direct-acting antivirals; NRTI, nucleoside analog HIV reverse; NNRTI, non-nucleoside analog HIV reverse transcriptase inhibitor; PI, protease inhibitor; II, integrase inhibitor; AIDS, acquired immune deficiency syndrome; HCV-RNA, HCV plasma viral load; LSM, liver stiffness measures; CPT, Child-Pugh-Turcotte; KVPG, hepatic venous pressure gradient (mmHg); a.u., arbitrary units of fluorescence; sCD14, soluble CD14; LPS, lipopolysaccharide; FABP2, fatty acid-binding protein 2; LBP, lipopolysaccharide binding protein; IL, interleukin; IL-1RA, interleukin-1 receptor antagonist; TNF-α, tumor necrosis factor alpha; IP-10, IFN-γ-inducible protein 10; MCP1, monocyte chemoattractant protein-1; OPG, osteoprotegerin; sRANKL, soluble receptor activator of nuclear factor-kappa B ligand; sVCAM-1, soluble vascular cell adhesion molecule 1; sICAM-1, soluble intercellular cell adhesion molecule 1; sTNFR-1, soluble tumor necrosis factor receptor 1; PAI-1, plasminogen activator inhibitor-1; VEGF-A; vascular endothelial growth factor A; sVEGF-R1, soluble receptor1 for vascular endothelial growth factor*.

### Change in Outcome Measures After Achieving SVR

The changes in the outcome measures during the follow-up are shown in Table 2. We found significant decreases in severity scores of liver disease [LSM (*q*-value < 0.001), HVPG (*q*-value = 0.011), and CTP (*q*-value = 0.045)] and plasma biomarkers [LBP (*q*-value < 0.001), IP-10 (*q*-value < 0.001), IL-8 (*q*-value < 0.001), IL-18 (*q*-value < 0.001), IL-1RA (*q*-value = 0.013), OPG (*q*-value < 0.001), sICAM-1 (*q*-value < 0.001), sVCAM-1 (*q*-value < 0.001), PAI-1 (*q*-value = 0.001), and VEGF-A (*q*-value = 0.006)].

**Table 2:** Change in severity scores of liver disease and plasma biomarkers during follow-up in HIV-infected patients with advanced HCV-related cirrhosis.

	**Δx (95%CI)**	***p***	***q***
**Liver disease**			
LSM (kPa)	−9.1 (−12.4; −5.8)	** <0.001**	** <0.001**
HVPG (mmHg)	−2.6 (−4.4; −0.8)	**0.005**	**0.011**
CTP score	−0.2 (−0.3; 0)	**0.024**	**0.045**
**Bacterial translocation**			
LPS (EU/ml)	0.1 (−0.2; 0.4)	0.372	0.431
LBP (μg/ml)	−0.6 (−0.8; −0.4)	** <0.001**	** <0.001**
sCD14 (μg/ml)	0.1 (−0.3; 0.5)	0.589	0.643
FABP-2 (ng/ml)	0.1 (−0.1; 0.3)	0.291	0.388
**Inflammatory response**			
IP-10 (a.u.)	−699.5 (−994.7; −404.3)	** <0.001**	** <0.001**
MCP-1 (a.u.)	−47.3 (−152.4; 57.7)	0.377	0.431
IL-8 (a.u.)	−55.9 (−80.9; −31)	** <0.001**	** <0.001**
IL-1β (a.u.)	−2.4 (−5.4; 0.6)	0.116	0.186
IL-18 (a.u.)	−901.2 (−1,300; −488.7)	** <0.001**	** <0.001**
IL-6 (a.u.)	−12.5 (−35.2; 10.2)	0.280	0.388
TNF-α (a.u.)	−1 (−2.6; 0.6)	0.228	0.341
IL-1RA (a.u.)	−11.9 (−20.4; −3.3)	**0.006**	**0.013**
sRANKL (a.u.)	−0.6 (−4.3; 3.1)	0.746	0.779
OPG (a.u.)	−82.7 (−116.1; −49.2)	** <0.001**	** <0.001**
**Endothelial dysfunction**			
sVCAM-1 (a.u.)	−1,400 (−2,100; −786.4)	** <0.001**	** <0.001**
sICAM-1 (a.u.)	−74.3 (−95.5; −53.1)	** <0.001**	** <0.001**
TNFR-I (a.u.)	−9 (−17.8; −0.2)	**0.044**	0.076
**Coagulopathy**			
PAI-1 (a.u.)	−468 (−733; −203)	**0.001**	**0.001**
D-dimer (a.u.)	547.4 (−511.3; 1,606.1)	0.311	0.393
**Angiogenesis**			
VEGF-A (a.u.)	−18.2 (−30; −6.4)	**0.003**	**0.006**
VEGF-R1 (a.u.)	0 (−6.9; 6.9)	0.999	0.999

*Statistics: Values are expressed as the estimated average of the increase (Δx = x2-x1) and 95% of confidence interval (95%CI). p-Values were calculated by GLM mixed models. p-Values, raw p-values; q-values, p-values corrected for multiple testing using the false discovery rate (FDR) with Benjamini and Hochberg procedure. The statistically significant differences are shown in bold*.

*HCV, hepatitis C virus; HIV, human immunodeficiency virus; LSM, liver stiffness measurement; CTP, Child-Pugh-Turcotte; HVPG, hepatic venous pressure gradient; mmHG, millimeter of mercury; kPa, kilopascals; a.u., arbitrary units of fluorescence; sCD14, soluble CD14; LPS, lipopolysaccharide; FABP2, fatty acid-binding protein 2; LBP, lipopolysaccharide binding protein; IL, interleukin; IL-1RA, interleukin-1 receptor antagonist; TNF-α, tumor necrosis factor alpha; IP-10, IFN-γ-inducible protein 10; MCP1, monocyte chemoattractant protein-1; OPG, osteoprotegerin; sRANKL, soluble receptor activator of nuclear factor- kappaB ligand; sVCAM-1, soluble vascular cell adhesion molecule 1; sICAM-1, soluble intercellular cell adhesion molecule 1; sTNFR-1, soluble tumor necrosis factor receptor 1; PAI-1, plasminogen activator inhibitor-1; VEGF-A; vascular endothelial growth factor A; sVEGF-R1, soluble receptors for vascular endothelial growth factor*.

**Figure 1:**
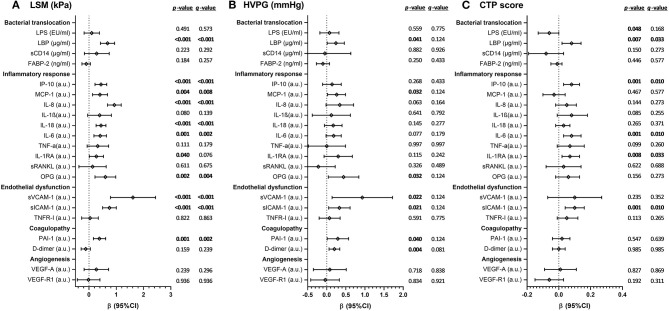
Association between the change in values of plasma biomarkers and severity scores of liver disease [**A**, LSM (kPa); **B**, HVPG (mmHg); **C**, CTP score] during HCV treatment in HIV/HCV-coinfected patients with advanced cirrhosis. Statistics: Data were calculated by GLM mixed models. Values are expressed as regression coefficient (β) and 95% of confidence interval (95%CI). *p-Values*, raw *p*-values; *q*-values, *p*-values corrected for multiple testing using the false discovery rate (*FDR*) with Benjamini and Hochberg procedure. The statistically significant differences are shown in bold. HCV, hepatitis C virus; HIV, human immunodeficiency virus; LSM, liver stiffness measurement; CTP, Child-Pugh-Turcotte; HVPG, hepatic venous pressure gradient; mmHg, millimeter of mercury; kPa, kilopascals; a.u., arbitrary units of fluorescence; sCD14, soluble CD14; LPS, lipopolysaccharide; FABP2, fatty acid-binding protein 2; LBP, lipopolysaccharide binding protein; IL, interleukin; IL-1RA, interleukin-1 receptor antagonist; TNF-α, tumor necrosis factor alpha; IP-10, IFN-γ-inducible protein 10; MCP1, monocyte chemoattractant protein-1; OPG, osteoprotegerin; sRANKL, soluble receptor activator of nuclear factor-kappa B ligand; sVCAM-1, soluble vascular cell adhesion molecule 1; sICAM-1, soluble intercellular cell adhesion molecule 1; sTNFR-1, soluble tumor necrosis factor receptor 1; PAI-1, plasminogen activator inhibitor-1; VEGF-A; vascular endothelial growth factor A; sVEGF-R1, soluble receptor1 for vascular endothelial growth factor.

Figure 1 shows the associations between the change in values of plasma biomarkers and severity scores of liver disease. A significant direct association between the change in LSM values and the change in values of LBP (*q*-value < 0.001), IP-10 (*q*-value < 0.001), MCP-1 (*q*-value = 0.008), IL-8 (*q*-value < 0.001), IL-18 (*q*-value < 0.001), OPG (*q*-value = 0.004), sVCAM-1 (*q*-value < 0.001), sICAM-1 (*q*-value < 0.001), and PAI-1 (*q*-value = 0.002) was found. For HVPG values, we found no significant association after the *p*-values were corrected for multiple testing. For CTP values, we found significant positive associations with IP-10 (*q*-value = 0.010), IL-6 (*q*-value = 0.010), IL-1RA (*q*-value = 0.033), and sICAM-1 (*q*-value = 0.010).

## Discussion

Both HIV and HCV infection and advanced cirrhosis promote inflammation, immune activation, and dysfunction of the immune system, which are all linked to greater severity of liver disease and the development of comorbidities (1–3). Bacterial translocation in patients with advanced HCV-related cirrhosis is a crucial step in the pathogenesis of spontaneous bacterial peritonitis and bacteremia, as well as a critical factor that triggers the immune activation and inflammation, which in turn promotes hemodynamic changes and the development of decompensated cirrhosis (3). Inflammation promotes endothelial dysfunction, which is linked to the progression of CHC and the development of cardiovascular events (38). Moreover, hepatocytes release most of the blood's proteins, where plasma levels can be altered by CHC progression, promoting an increase in thrombotic risk (39). Coagulopathy has been related to a higher risk of disease progression and death in HIV-infected patients (40) and patients with advanced liver disease (41). A recent article on HIV/HCV-coinfected patients showed that plasma biomarkers of bacterial translocation, inflammation, endothelial dysfunction, and coagulopathy increased with increased liver fibrosis severity, particularly in patients with LSM ≥ 40 KPa (35). Besides, plasma IP-10 and IL-6 have also been correlated to advanced liver disease (CTP 7-9) (42). However, the eradication of HCV with antiviral therapy can stop this pathological process of the liver, at least almost entirely (34). Additionally, the decrease in inflammation biomarker levels could also indicate a lower risk of developing comorbidities in cirrhotic patients who achieved SVR with DAA therapy (1, 24).

In our study, HCV clearance after DAA therapy promoted a significant improvement in severity scores of liver cirrhosis and many plasma biomarkers linked to inflammation (bacterial translocation, inflammatory response, and endothelial dysfunction) and coagulopathy. Our data are in concordance with many previous studies that found a significant decrease in liver disease scores of HIV/HCV-coinfected patients (6, 7, 31, 43–45) and HCV-monoinfected patients (6–14) after HCV eradiation with DAA therapy. Besides, significant decreases have been found in plasma biomarkers of HIV/HCV-coinfected patients (18, 29–33) and HCV-monoinfected patients (15–20). However, there is an important lack of consistency in these previous publications regarding the plasma biomarkers and liver severity scores evaluated, time-points used to take data or samples after the end of HCV treatment, statistical analysis used, and liver fibrosis stages included.

Liver injury leads to the secretion of VEGF-A that activates quiescent HSCs into proliferative, contractile, and fibrogenic myofibroblasts (activated HSCs; aHSCs) involved in collagen deposition and development of cirrhosis (46). VEGF-A plays a crucial role in liver cancer and higher circulating VEGF-A levels correlate with tumor angiogenesis, rapid disease progression, and reduced survival (47). Besides, high VEGF-A levels are related to portal hypertension (48). Therefore, a decrease in VEGF-A values indicates a good prognosis for liver disease. Previous reports did not find any change in plasma VEGF-A values after HCV eradication with DAA therapy (43, 49) or showed a significant increase in HCV-monoinfected patients (50). In our study, we found a reduction in plasma VEGF-A levels after HCV clearance with HCV therapy, suggesting discontinuation in the process of angiogenesis. However, this decrease in plasma VEGF-A levels was not associated with changes in liver cirrhosis severity scores. In this regard, it would have been helpful to have other biomarkers of angiogenesis and fibrosis (47), such as platelet-derived growth factor (PDGF), transforming growth factor-β1 (TGF-β1), and fibroblast growth factor (FGF), but these were not measured in our study.

The relationship between plasma biomarkers (bacterial translocation, inflammatory response, endothelial dysfunction, coagulopathy, and angiogenesis) and liver disease scores (LSM, HVPG, and CTP) has been scarcely explored in patients with advanced cirrhosis after HCV eradication with DAAs. Laursen et al. (20) reported that the levels of macrophage activation markers (sCD163 and soluble mannose receptor) correlated with LSM values during follow-up in HCV-monoinfected patients. Kostadinova et al. (31) showed that an improvement in LSM values associated with a decrease in sCD14 levels after IFN-free HCV therapy. Additionally, plasma levels of sCD163, IL-6, and Mac2-binding protein correlated with AST level and APRI score changes. In our study, decreases in several plasma biomarkers levels linked to bacterial translocation, inflammatory response, endothelial dysfunction, and coagulopathy were directly associated with the reductions in liver disease scores (LSM and CTP) after HCV eradication with DAAs. Therefore, our data suggest a relation between improvement in liver cirrhosis and resolution of inflammation after HCV eradication with all-oral DAAs.

This research has several strengths and limitations that must be discussed. Firstly, the sample size was limited, which may negatively affect this study's statistical power and mask some significant values. Despite this, it should be noted that our study was multicenter, prospective, and only included patients with advanced cirrhosis. Secondly, the variation in plasma biomarkers was assessed at baseline and 48 weeks after completing HCV therapy (repeated measure design) and was analyzed by GLM mixed (each patient is your control), a statistical analysis that gives robustness to our data. Thirdly, the number of plasma biomarkers evaluated was large, and despite adjustment for multiple comparisons and the small sample size, many biomarkers were found to have significant *p*-values. Finally, the outcomes in the intermediate clinical time points, such as during DAA therapy, end of treatment (EOT), and SVR, were not collected. These intermediate time points may provide relevant information. However, we aimed to examine only the outcomes at baseline (pre-DAA therapy) and the end of follow-up (post-SVR) because it was a simple design that could facilitate the achievement of our objectives.

## Conclusion

In conclusion, the HCV eradication with all-oral DAAs in HIV/HCV-coinfected patients with advanced cirrhosis promoted an improvement in the severity of advanced cirrhosis and plasma biomarkers (inflammation, coagulopathy, and angiogenesis). The decrease in plasma biomarkers was mainly related to the reduction in LSM values.

## Data Availability Statement

The raw data supporting the conclusions of this article will be made available by the authors, without undue reservation.

## Ethics Statement

The studies involving human participants were reviewed and approved by Ethics Committee of the Instituto de Salud Carlos III (CEI PI 41_2014). The patients/participants provided their written informed consent to participate in this study.

## Author Contributions

SR, JB, and JG-G: conceptualization. JB, JG-G, CD, VH, PG-B, and LI-S: data curation. LM, SS, JM, SR, and MAJ-S: formal analysis. JB, JG-G, MAJ-S, and SR: funding acquisition. LM and PG-B: investigation and methodology. JB: project administration. SR: supervision and visualization. MAJ-S and SR: writing—original draft preparation. LM and JB: writing—review and editing. All authors have read and approved the final manuscript.

## Conflict of Interest

The authors declare that the research was conducted in the absence of any commercial or financial relationships that could be construed as a potential conflict of interest.

## Abbreviations

HCV: hepatitis C virus
CHC: chronic hepatitis C
CAID: cirrhosis-associated immune dysfunction
DAAs: direct-acting antiviral agents
SVR: Sustained virologic response
LSM: liver stiffness measurement
HVPG: hepatic liver pressure gradient
CTP: Child-Pugh-Turcotte
HIV: human immunodeficiency virus
ART: combination antiretroviral therapy
kPa: kilopascals
mmHG: millimeter of mercury
sCD14: soluble CD14
LPS: lipopolysaccharide
FABP2: fatty acid-binding protein 2
LBP: lipopolysaccharide binding protein
IP-10: IFN-γ-inducible protein 10
MCP1: monocyte chemoattractant protein-1
IL: Interleukin
TNF-α: tumor necrosis factor alpha
IL-1RA: interleukin-1 receptor antagonist
sRANKL: soluble receptor activator of nuclear factor-kappa B ligand
OPG: osteoprotegerin
sVCAM1: soluble vascular cell adhesion molecule 1
sICAM1: soluble intercellular cell adhesion molecule 1
sTNFR1: soluble tumor necrosis factor receptor 1
PAI-1: plasminogen activator inhibitor-1
VEGF-A: vascular endothelial growth factor A
sVEGF-R1: soluble receptor 1 for vascular endothelial growth factor
GLMM: Generalized Linear Mixed Models
IFN: interferon.

## Appendix

**The ESCORIAL study group**

**Hospital General Universitario Gregorio Marañón** (Madrid, Spain): Cristina Díez, Luis Ibáñez, Leire Pérez-Latorre, Diego Rincón, Teresa Aldámiz-Echevarría, Vega Catalina, Pilar Miralles, Teresa Aldámiz-Echevarría, Francisco Tejerina, María C Gómez-Rico, Esther Alonso, José M Bellón, Rafael Bañares, and Juan Berenguer.

**Hospital Universitario La Paz/IdiPAZ** (Madrid, Spain): José Arribas, José I Bernardino, Carmen Busca, Javier García-Samaniego, Víctor Hontañón, Luz Martín-Carbonero, Rafael Micán, María L Montes-Ramírez, Victoria Moreno, Antonio Olveira, Ignacio Pérez-Valero, Eulalia valencia, and Juan González-García.

**Hospital Universitario Puerta de Hierro** (Madrid, Spain): Elba Llop and José Luis Calleja.

**Hospital Universitario Ramón y Cajal** (Madrid, Spain): Javier Martínez and Agustín Albillos.

**Fundación SEIMC/GeSIDA** (Madrid, Spain): Marta de Miguel, María Yllescas, and Herminia Esteban.
